# Hypoxia dampens innate immune signalling at early time points and increases Zika virus RNA levels in iPSC-derived macrophages

**DOI:** 10.1099/jgv.0.001885

**Published:** 2023-08-16

**Authors:** Mirjam Schilling, Alun Vaughan-Jackson, William James, Jane A. McKeating

**Affiliations:** ^1^​ Nuffield Department of Clinical Medicine, University of Oxford, Oxford OX3 7FZ, UK; ^2^​ James & Lillian Martin Centre, Sir William Dunn School of Pathology, University of Oxford, South Parks Road, Oxford OX1 3RE, UK

**Keywords:** hypoxia, IFN, ISGs, MX1, RIG-I, ZIKV, iPSC, HIF

## Abstract

Type I interferons (IFNs) are the major host defence against viral infection and are induced following activation of cell surface or intracellular pattern recognition receptors, including retinoic-acid-inducible gene I (RIG-I)-like receptors (RLRs). All cellular processes are shaped by the microenvironment and one important factor is the local oxygen tension. The majority of published studies on IFN signalling are conducted under laboratory conditions of 18% oxygen (O_2_), that do not reflect the oxygen levels in most organs (1–5 % O_2_). We studied the effect of low oxygen on IFN induction and signalling in induced Pluripotent Stem Cell (iPSC)-derived macrophages as a model for tissue-resident macrophages and assessed the consequence for Zika virus (ZIKV) infection. Hypoxic conditions dampened the expression of interferon-stimulated genes (ISGs) following RLR stimulation or IFN treatment at early time points. RNA-sequencing and bio-informatic analysis uncovered several pathways including changes in transcription factor availability, the presence of HIF binding sites in promoter regions, and CpG content that may contribute to the reduced ISG expression. Hypoxic conditions increased the abundance of ZIKV RNA highlighting the importance of understanding how low oxygen conditions in the local microenvironment affect pathogen sensing and host defences.

## Introduction

Successful virus infection is dependent on the presence of specific cellular host factors and evasion of both innate and adaptive immune responses. The cellular microenvironment can affect a multitude of pathways that impact virus replication. Recent studies from our laboratory have identified a role for oxygen tension in regulating cellular susceptibility to virus infection (reviewed in [1]). Depending on the blood supply and metabolic demand the oxygen tension can vary between 1 and 5 % in different organs [2]. This contrasts with many reported studies that employ *in vitro* tissue culture model systems that are maintained at 18 % oxygen (O_2_). Lower oxygen conditions, referred to as hypoxia, will inactivate multiple oxygen sensing mechanisms, including the prolyl hydroxylases (PHD) and factor inhibiting HIF (FIH)-hypoxia inducible factor (HIF) pathways that stabilise HIF expression [3, 4]. Hypoxia can modulate the replication of a wide number of viruses [1], where HIFs enhance the replication of hepatitis B [5] and Epstein Barr viruses [6, 7] via direct binding to their viral DNA genomes. In contrast, a hypoxic environment suppresses influenza A virus [8], SARS-CoV-2 [9, 10] and HIV-1 [11] replication. These differing outcomes may reflect variable oxygen levels at the site of virus replication.

The type I IFN system is a crucial first line of defence to combat pathogens [12]. Pathogen associated molecular patterns (PAMPs) are recognized by cellular sensors, such as Toll like receptors (TLRs), RIG-I like receptors (RLRs) or cytoplasmic DNA receptors. Through downstream activation of kinases and transcription factors, IFNs are induced and stimulate the expression of interferon-stimulated genes (ISGs) that create an antiviral state. A recent study reported that hypoxia downregulates the RLR dependent type I IFN pathway in cancer cell lines and this associated with a decreased accessibility of STAT1 and IRF3 motifs in host chromatin [13]. Since RLR signalling is a key inducer of innate immune responses we chose to study the consequences of low oxygen on IFN induction and signalling in primary immune cells. We found that hypoxia dampened ISG expression in human induced pluripotent stem cell (iPSC) derived macrophages early after stimulation with RLR activators and IFN. RNA-Seq analysis suggests this is mediated by a dysregulation of pathways that affect the expression and stability of ISG transcripts, including changes in transcription factor availability, the presence of HIF binding sites in promoter regions, and CpG content.

RIG-I is the main sensor that detects Zika virus (ZIKV) [14–19], a member of the *Flaviviridae* family, that was first isolated from sentinel rhesus macaque in the Zika Forest in Uganda and later from *Aedes africanus* mosquitoes [20]. Despite causing a self-limiting acute febrile illness in adults, ZIKV infection during the first trimester of pregnancy is associated with multiple neurodevelopmental defects, including microcephaly in newborns [21]. ZIKV has been reported to infect Hofbauer cells (HBCs), placental macrophages, in isolated cultures and placental explants (reviewed in [22]). ZIKV antigen was found in HBCs in placental tissue retrieved from a miscarriage of a confirmed ZIKV-infected mother in a Brazilian case study [23]. HBCs were reported to express pro-inflammatory cytokines and type I interferon in response to ZIKV infection and may provide a protective response against the virus. Developmentally, HBCs are a diverse group of cells with multiple origins depending on the gestational stage. During early stages of pregnancy, they are thought to originate from the hypoblast-derived yolk sac and are similar to tissue-resident macrophages, such as microglia, Kupffer cells, and Langerhans cells, which derive from Myb-independent yolk sac (YS) progenitors. We selected iPSC-derived macrophages as a model to study the effect of hypoxia on ZIKV infection as they also develop in an MYB-independent, runt-related transcription factor 1 (RUNX1)-, and Spleen Focus Forming Virus (SFFV) Proviral Integration Oncogen (SPI1)-dependent fashion and provide a model for MYB-independent tissue-resident macrophages [24].

## Methods

### Cells and reagents

The iPSC-derived macrophages were differentiated from the human iPSC line OX1-61 [25, 26] and cultured in advanced DMEM/F12 supplemented with 1 % penicillin/streptomycin, Glutamax (2 mM), stabilized Insulin (5 µg ml^−1^), HEPES pH 7.4 (15 mM), M-CSF (100 ng ml^−1^). The human iPSC derived lines used in this study are SFC841-03-01 [27] (donor #1) and SFC840-03-03 [28] (donor #2).

**Table:** 

*Reagent*	*Source*	*Catalogue no.*
Poly(I:C)	Sigma-Aldrich	P1530
5’pppRNA	InvivoGen	tlrl-3prna
IFNα2a	PeproTech	300-02AA

### Viruses

The Brazilian ZIKV isolate ZIKV/H. sapiens/Brazil/PE243/2015 was originally described in [29] and was propagated in Vero cells. Infectivity of viral stocks was determined by plaque assay using A549 BVDV NPro cells. These cells stably express the NPro protein of bovine viral diarrhoea virus (BVDV), which induces degradation of IRF3, and are optimized for virus growth [30].

### Cell viability assay

Viability was assessed using the CellTiter-Fluor Cell Viability Assay (Promega, G6080) according to manufacturer’s instructions. Fluorescence was measured on the Spectramax M5 plate reader using SoftmaxPro software version 5.

### qRT-PCR

Cells were lysed and total RNA extracted using the RNeasy kit (Qiagen) according to the manufacturer’s instructions. Equal amounts of cDNA were synthesized using the High Capacity cDNA Kit (Applied Biosystems) and mRNA expression determined using Fast SYBR master mix in a StepOne thermocycler (Applied Biosystems). C_T_ values were normalized to TBP (ΔC_T_). SYBR green primer probes used include TBP (for: CCCATGACTCCCATGACC, rev: TTTACAACCAAGATTCACTGTGG), NDRG1 (for: TTTGATGTCCAGGAGCAGGA, rev: ATGCCGATGTCATGGTAGGT), MX1 (for: GGCTGTTTACCAGACTCCGACA, rev: CACAAAGCCTGGCAGCTCTCTA), DDX58 (for: CACCTCAGTTGCTGATGAAGGC, rev: GTCAGAAGGAAGCACTTGCTACC) and ZIKV (for: TCGTTGCCCAACACAAG, rev: CCACTAATGTTCTTTTGCAGACAT).

### Western blotting

Cells were lysed in RIPA buffer (20 mM Tris pH 7.5, 2 mM EDTA, 150 mM NaCl, 1 % NP40, 1 % SDS, 0.1 % TritonX100, 0.25 % Na-Deoxychalate, plus protease inhibitor) and the samples were incubated at 95 °C for 5 min. Protein lysates were separated on 10 % SDS-PAGE gels and transferred onto polyvinylidene difluoride (PVDF) membranes. As primary antibodies we used anti-Mx (mouse, M143 [31]), anti-RIG-I (mouse, AdipoGen), and anti-beta-actin (mouse, AC-15, Sigma-Aldrich). Primary antibodies were detected with peroxidase-conjugated secondary antibodies (GE Healthcare).

### Flow cytometry analysis

To measure cell viability, cells were stained with Live Dead Aqua (Life Technologies, UK) in addition to the primary antibody (IFNAR1, EP899Y, rabbit, abcam). After washing, cells were stained with secondary antibody (goat anti-rabbit, Alexa Fluor 633) and resuspend in BD Cell FIX. Samples were acquired on a Cyan ADP flow cytometer (Beckman Coulter) and data analysed using FlowJo (TreeStar).

### RNA-Seq and bio-informatic analysis

RNA was isolated from three samples per condition (1 % O_2_ and 18 % O_2_) using the RNeasy kit (Qiagen) and RNA-sequencing performed by Novogene (Illumina): RNA purity was assessed with a NanoDrop 2000 spectrophotometer (Thermo Fisher Scientific) and integrity determined using a 2100 Bioanalyzer Instrument (Agilent Technologies). Sequence adapters, reads containing ploy-N and low quality reads were removed from the raw data through in-house perl scripts. The reads were mapped against the reference human genome (GRCh38/hg38) using hisat2 v2.0.5. Counts per gene were calculated using featureCounts v1.5.0-p3 and the FPKM of each gene was calculated based on the length of the gene and reads count mapped to this gene. Reads were analysed by edgeR v3.22.05. The *P* values were adjusted using the Benjamini and Hochberg method. Corrected *P*-value of 0.05 and absolute foldchange of two were set as the threshold for significant changes in gene expression. Raw read files available through NCBI (PRJNA976211).

Gene set enrichment analysis (GSEA) was performed using GSEA_4.1.0, and hallmark gene sets retrieved from the Molecular Signatures Database (MSigDB) [32, 33]. As permutation-type we used ‘gene_set’, and as cut-off an FDR of 0.25. GraphPad Prism (version 9.3.1, GraphPad, San Diego, CA, USA) was used to plot the normalized enrichment score (NES) and colour-code by the false discovery rate (FDR). Transcription factor binding sites of HIF1A (ranging from −1000 to 100 bp relative to TSS, with cut-off *P*=0.001) were analysed using https://epd.epfl.ch//index.php. Transcription factor enrichment analysis (TFEA) was performed through the ChEA3 background database (https://maayanlab.cloud/chea3/) to compare discrete query gene sets with libraries of target gene sets assembled from multiple orthogonal 'omics' datasets [34].

Dinucleotides were counted using R (version 4.0.3 [2020-10-10]) in Rstudio (Version 1.4.1103). Sequences were downloaded from the NCBI database (Genbank), dinucleotides counted for all 16 combinations and normalized to the length of the respective sequence [35]. CpG content of up- and downregulated ISGs, or the top 30 up- and downregulated protein-coding genes based on log2-fold or adjusted *P*-value were plotted with GraphPad Prism (version 9.3.1, GraphPad, San Diego, CA, USA) and a nonparametric t-test (Mann-Whitney) was performed, *P<0.05, **P<0.001.

### Quantification and statistical analysis

Data were analysed using GraphPad Prism version 9.3.1 (GraphPad, San Diego, CA, USA). All data are presented as mean values ± SEM. Significance values are indicated as ∗*P*<0.05; ∗∗*P*<0.01; ∗∗∗*P*<0.001; ∗∗∗∗*P*<0.0001. n.s. denotes non-significant. Please see individual figure legends for further details.

## Results

### Hypoxia dampens ISG transcripts but not IFNAR1 cell surface expression

iPSC-derived macrophages were differentiated from pluripotent stem cells via embryoid body intermediates as previously reported [26]. This protocol uses open-source media components that result in a lower basal ISG expression compared to cells differentiated using earlier methods, while being more responsive to inflammatory stimulation. We used independent differentiations of stem cells from the same donor, or a second, independent donor.

**Fig. 1.**
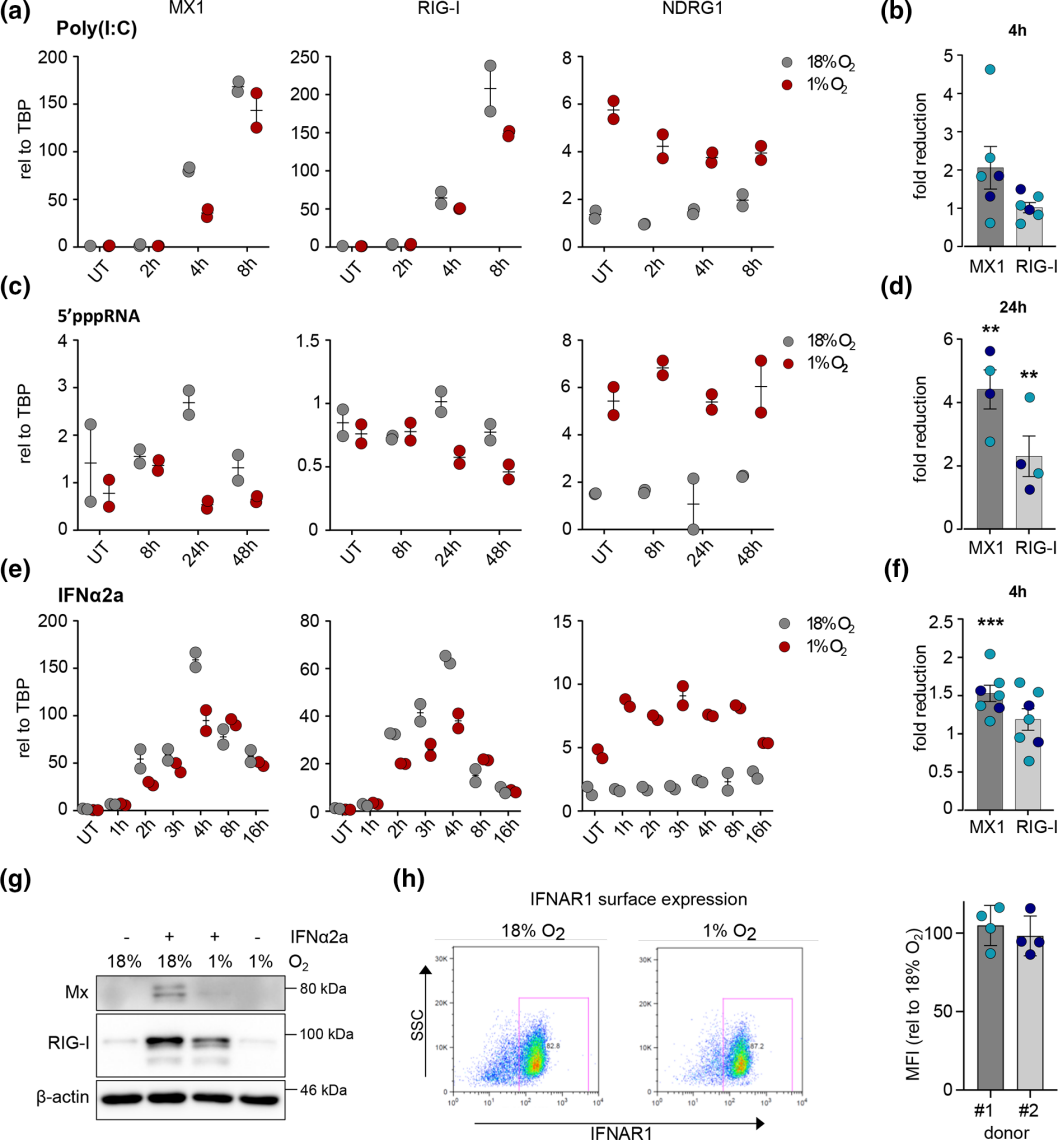
Hypoxia dampens ISGs but not IFNAR1 cell surface expression. RT-qPCR analysis of iPS-derived macrophages. (a) transfected with Poly(I:C) (0.2 µg ml^−1^) 24 h after incubating under normoxic (18 % oxygen) or hypoxic (1 % oxygen) conditions. One representative experiment of donor #1 with two technical replicates is shown. (**b**) Two independent differentiations of donor #1 (teal) with *n*=4 independent experiments, plus *n*=2 experiments with donor #2 (dark blue) relative to normoxia are shown for one timepoint. (**c**) transfected with 5’pppRNA (0.2 µg ml^−1^) 24 h after incubating under normoxia or hypoxia. One representative experiment of donor #1 with two technical replicates is shown. (**d**) One independent differentiation of donor #1 (teal) with *n*=2 and donor #2 (dark blue) with *n*=2 relative to normoxia is shown for one timepoint. (**e**) treated with IFNα2a (100 U ml^−1^) 24 h after exposure to normoxia or hypoxia. One representative experiment of donor #1 with two technical replicates is shown. (**f**) Two independent differentiations of donor #1 (teal) with *n*=5, plus *n*=2 experiments with donor #2 (dark blue). Unpaired t-test with *P<0.05, **P<0.01., ***P<0.001. (**g**) Western blot analysis of donor#1 exposed to normoxia or hypoxia for 24 h followed by a 24 h treatment with IFNα2a (100 U ml^−1^). One representative blot (*n*=3) is shown. (**h**) Flow cytometric analysis of IFNAR1 cell surface expression after 24 h of hypoxia. One representative FACS plot is shown for donor#1 and the MFI normalized to 18 % for *n*=3 independent experiments for both donors. UT, untreated.

To investigate the effect of hypoxia on RLR signalling iPSC-derived macrophages were cultured at 1 % O_2_ or 18 % O_2_ for 24 h and transfected with Poly(I:C) (Fig. 1A). Poly(I:C) is a synthetic mimetic of a double-stranded RNA that is detected by TLRs or, when transfected, by RLRs. We selected a concentration of Poly(I:C) that did not affect cell viability and induced ISG expression (Fig. S1, available in the online version of this article). We measured expression of MX Dynamin Like GTPase 1 (*MX1*), an ISG solely induced by type I and type III IFNs, and *RIG-I,* which can be directly stimulated by Interferon regulatory factor 3 (IRF3) activation. As a control we measured transcript levels of the HIF regulated gene N-Myc Downstream Regulated 1 (*NDRG1*) (Fig. 1a, c, e). While hypoxia induced *NDRG1* gene expression, we observed a modest reduction in *MX1* and *RIG-I* transcripts. Studying cells at 4 h post-Poly(I:C) treatment from an independent differentiation along with cells from an additional donor showed a two-fold reduction in *MX1* gene and a modest change in *RIG-I* gene expression (Fig. 1b). To specifically stimulate RIG-I the cells were transfected with 5’pppRNA [36] and we observed a reduction in *MX1* and *RIG-I* gene expression at the peak of gene induction (Fig. 1c). Repeat experiments showed a significant 2–4 fold reduction in *MX1* and *RIG-I* transcripts (Fig. 1d). To differentiate between the effects on intracellular RNA sensing and secondary intercellular IFN signalling, we treated the iPSC-derived macrophages with IFNα2a and observed a reduction in *MX1* and *RIG-I* gene expression at 4 h post-stimulation (Fig. 1e, f). This reduction in ISG expression waned at later time points, similar to our observations after Poly(I:C) transfection. The hypoxic suppression of *MX1* and *RIG-I* transcripts following IFN treatment (24 h) was more pronounced at the protein level, with Mx protein expression reduced 7- to 11-fold (Fig. 1g).

One explanation for the observed dampening in ISG expression following Poly(I:C) and 5’pppRNA treatment may be a reduction in Interferon Alpha And Beta Receptor Subunit 1 (IFNAR1) expression. To examine this, we stained IFNAR1 on iPSC-derived macrophages cultured at 18 % or 1 % O_2_ for 24 h. IFNAR1 surface expression was unchanged in hypoxic conditions compared to normoxia (Fig. 1h and Fig. S2). Overall, these findings show that changes in the local oxygen tension affect RIG-I like receptor sensing and IFN signalling in iPSC-derived macrophages that result in a dampened ISG response.

**Fig. 2.**
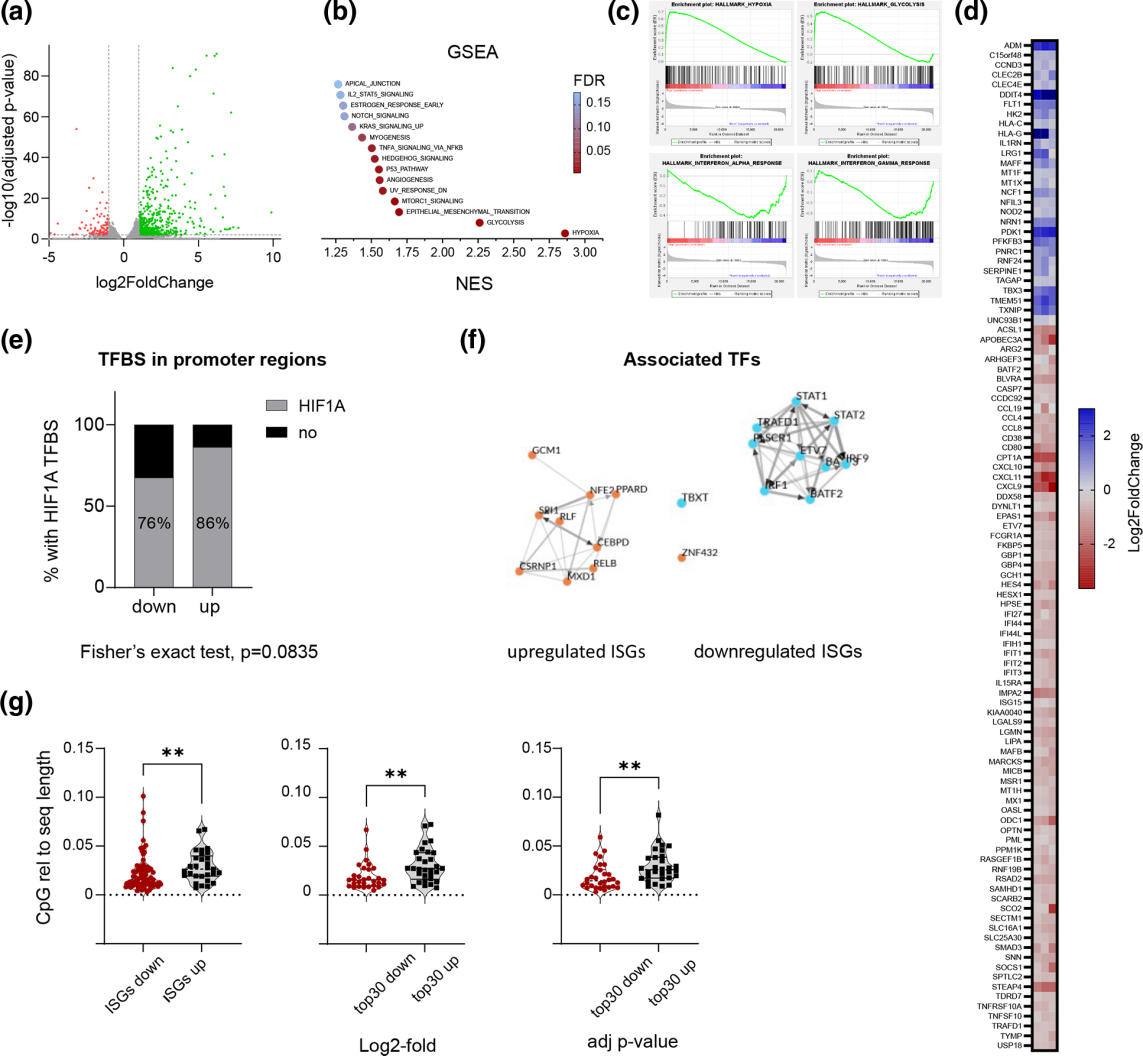
RNA-Seq analysis of IFN-treated iPSC-derived macrophages in hypoxia reveals a broad dampening of IFNα and IFNγ hallmark genes. (**a**) Volcano plot showing the distribution of differentially expressed genes between cells in hypoxia vs normoxia of donor #1 4 h after IFNα2a stimulation. (**b**) Gene set enrichment analysis using the hallmark gene sets from the MSigDB. Plots show the NES colour-coded by the FDR. (**c**) GSEA shows positive enrichment scores of gene sets associated with hypoxia or glycolysis, and negative enrichment scores of gene sets associated with IFNα or IFNγ responses. (**d**) Heatmap of 103 differentially expressed ISGs. (**e**) Bar graph representing the percentage of up- or downregulated ISGs with a ARNT::HIF1A transcription factor binding motif in their promoter region (−1000 relative to transcription start site). (**f**) TF enrichment analysis of TFs associated with up- or downregulated ISGs. Shown is the local network of the top ten TFs ranked across libraries. (**g**) CpG content relative to sequence length of the transcript of either the up- and downregulated ISGs, or the top 30 up- and downregulated protein-coding genes based on log2-fold or adjusted p-value. Nonparametric t-test (Mann-Whitney), *P<0.05, **P<0.01.

### Transcriptomic analysis of IFN-treated hypoxic iPSC-derived macrophages reveals a broad dampening of IFNα and IFNγ hallmark genes

To investigate whether hypoxia leads to global reduction in ISGs we performed a RNA-Seq analysis of IFN-treated iPSC-derived macrophages 4 h post-stimulation with IFNα2a. Three samples per condition (1 % O_2_ and 18 % O_2_) were analysed. A total of 510 genes were significantly up-, and 106 genes downregulated in hypoxia compared to normoxia (Fig. 2a). Gene set enrichment analysis (GSEA) showed a significant increase in genes connected to hypoxia and glycolysis confirming the cellular response to low oxygen (Fig. 2b, c). Additional pathways related to MTORC, TNFα, IL-2, Notch or Kras signalling were significantly increased. Importantly, GSEA confirmed that hypoxia reduced IFNα and IFNγ hallmark genes (Fig. 2c). We noted that not all of the 103 differentially expressed ISGs were suppressed under the hypoxic conditions (Fig. 2d). We found a total of 29 upregulated and 74 downregulated ISGs. Interestingly, many of the well-described direct anti-viral acting ISGs, such as *MX1*, Interferon-Induced Protein With Tetratricopeptide Repeats 1 (*IFIT1*) or SAM And HD Domain Containing Deoxynucleoside Triphosphate Triphosphohydrolase 1 (*SAMHD1*) were suppressed under the hypoxic conditions.

To investigate the mechanisms through which hypoxia affects ISG expression, we sought to identify pathways that could discriminate between the differentially expressed ISGs. We first analysed the presence or absence of transcription factor binding motifs in the respective promoter regions (−1000 relative to transcription start site) using the eukaryotic promoter database. Interestingly, the majority of promoter regions analysed encoded hypoxic response elements (HREs) (Fig. 2e). However, only about 10 % more of the upregulated ISGs encoded a HIF binding motif compared to the downregulated ISGs, suggesting this is unlikely to account for the differences in transcriptional regulation between the groups.

To gain a deeper insight into the transcriptional regulation of the ISGs we performed a transcription factor (TF) enrichment analysis to identify factors that associate with up- or downregulated ISGs using the ChIP-X Enrichment Analysis 3 (ChEA3) [34]. The predicted TFs associated with up- and downregulated ISGs are shown in Fig. 2f. We asked whether any of these key transcriptional regulators were differentially expressed under hypoxic conditions to account for the variable ISG expression. Comparing the predicted TFs encoded in the up- or downregulated genes identified several TFs associated with the upregulated ISGs, including CSRNP1, MXD1, PPARD, RLF and ZNF432. Additionally, the expression of BATF2, a TF associated with the downregulated group of ISGs was decreased. These data suggest that hypoxia causes an imbalance in the expression of several TFs that will impact the regulation of ISG subsets.

The level of transcripts in a cell is, however, not only affected by the expression or binding of transcription factors, but also by the availability of nucleotides. Our RNAseq analysis showed that hypoxia dysregulates nucleoside metabolism pathways (Fig. S3). This is in line with a reported depletion of nucleotides under hypoxia and an increase in the assembly of the multienzyme purinosome complex [37, 38]. Interestingly, Shaw *et al*. reported that ISGs have a lower CpG content than the human transcriptome [39]. We explored whether our two groups of ISGs differ in their dinucleotide content. Increased availability or lack of specific nucleotides potentially caused by a dysregulated nucleoside metabolism might well affect transcription. This could then lead to an increase or decrease of the expression of certain genes under hypoxia. Quantification of the dinucleotide content of our two ISG groups and showed that they differed significantly in their CpG content (Fig. 2g). To investigate whether this is a common pathway of ISG and host gene regulation under hypoxia we analysed the CpG content of the 30 most highly up- or downregulated genes in our dataset, based on either their 2log-fold or adjusted *P*-values (Fig. 2g). Interestingly, not only the up- and downregulated ISGs but also the 30 top hypoxic up- or downregulated host genes differed in their CpG content. This suggests that hypoxia may affect gene expression based on the dinucleotide content of transcripts.

Overall, our data show that hypoxia most likely affects the expression of ISGs through changes in transcription factor availability, the presence of HIF binding sites in promoter regions, and intrinsic properties of the transcripts, such as CpG content.

### Hypoxia promotes the abundance of ZIKV RNA in iPSC-derived macrophages

**Fig. 3.**
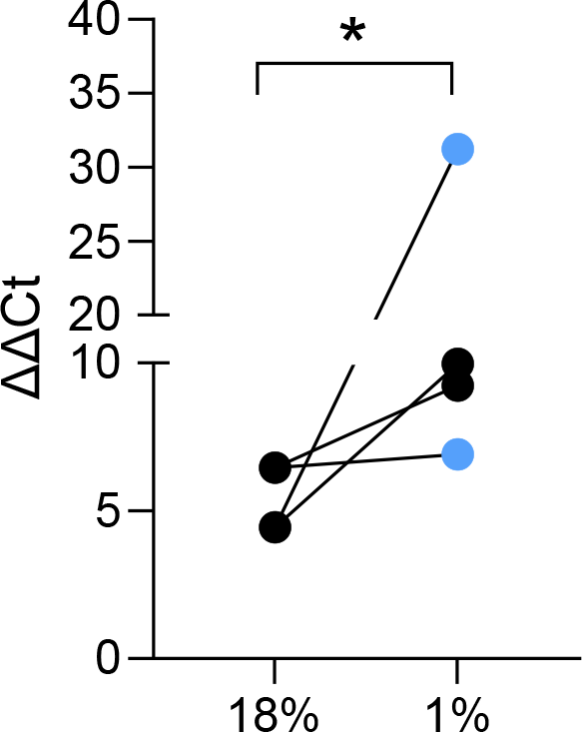
Hypoxia promotes ZIKV RNA levels in iPSC-derived macrophages. Comparison of ZIKV RNA levels in iPS-derived macrophages by RT-qPCR 24 h after infection (MOI 1) and culture at 18 % or 1 % O_2_. Cells were either maintained at 1 % O_2_ (pale blue) for 24 h before infection or at 18 % O_2_ (black) *n*=4 of one differentiation of donor #1. Nonparametric t-test (Mann-Whitney), *P<0.05.

Since our RNAseq analysis showed a suppression of IFNα and IFNγ hallmark genes, we speculated this would associate with an increase in virus replication. We chose to study ZIKV as it is mainly sensed by RIG-I [14–19]. To investigate the functional consequences of a dampened ISG response under hypoxia, we infected iPSC-derived macrophages kept in hypoxia or normoxia with ZIKV (MOI 1) and measured viral RNA levels 24 h after infection by RT-qPCR. We detected a significant increased in ZIKV RNA levels in the hypoxic iPSC-derived macrophages (Fig. 3). We compared both pre-incubation with 1 and 18 % O_2_ for 24 h before infection, because hypoxic signalling is dynamic. Interestingly, we found no significant difference in the increase in ZIKV replication between the conditions.

## Discussion

Our study shows that hypoxia dampens ISG expression in iPSC-derived macrophages at early times following stimulation, that are important in the initiation of rapid antiviral responses. In line with this, we detected increased levels of ZIKV RNA under hypoxia. Interestingly, we observed the most significant dampening of ISG expression after stimulation with IFNα2a, suggesting that both PAMP sensing as well as interferon signalling are affected by the changes in the oxygen tension. It is tempting to speculate that the higher fold-difference we detected in the sensing of 5’ppp RNA compared to IFN signalling reflects an additive effect of hypoxia on both IFN induction and IFN signalling. Our findings are consistent with a recent report describing that hypoxia suppresses the induction of type I IFN in monocytes [40] as well as in mouse peritoneal macrophages [41]. Furthermore, our results agree with earlier studies, reporting an increase in the replication of other members of the *Flaviviridae* family, such as hepatitis C and dengue viruses under low oxygen conditions [42–44].

Our findings may be relevant in the context of ZIKV infection *in vivo*, where oxygen levels in the placenta are approx 2–3 % in the first trimester and 5–8 % in the second and third trimesters of pregnancy [45]. ZIKV infection induces inflammation in the placenta [46], that may cause a further reduction of oxygen levels. The 1 % O_2_ we used in our experiments aligns with other reported studies that use this oxygen concentration to ensure HIF expression and to model inflamed tissue [9, 11, 47].

Our data suggest these conditions may contribute to the severe ZIKV-induced neurodevelopmental defects due to lower innate immune responses and higher ZIKV replication. *In vivo* experiments in mice show an increased permeability of the blood brain barrier 6 h after hypoxia. This could increase access of the virus to the embryonic brain during development, putting the embryo at higher risk to neurodevelopmental defects [48].

Hypoxia was reported to reduce host responses to TLR ligands in airway epithelia cells [49] in a HIF-1α-dependent manner. However, our data suggest that the presence or absence of HIF-1α-binding sites in the promoter regions of ISGs does not associate with the overall up- or downregulation observed. We recognise that bioinformatic predictions have limitations and this is reinforced by a recent HIF-1 and HIF-2 ChIP-Seq analysis that identified only 500–100 binding sites in the human genome (500–1000), despite their high abundance (over 1 million) [50, 51]. Transcriptional regulation is cell-type specific, emphasizing the importance of gaining experimental data through ChIP-Seq data to validate our bioinformatic analyses [52]. Apart from HIF-dependent changes other cellular pathways are activated under hypoxic conditions and will affect the transcriptome. Miar and colleagues reported a minimal role for HIF-1α or HIF-2α in regulating the hypoxic suppression of ISGs in cancer cell lines, but decreased chromatin accessibility of genomic regions relevant for type I IFN pathways [13]. Recent studies reported that lactate, a glycolytic product that is increased during hypoxia, regulates gene transcription via lactylation of histones [53, 54]. In tumor-infiltrating myeloid cells H3K18 lactylation increased N6-adenosine-methyltransferase 70 kDa subunit (*Mettl3*) expression that modified *Jak1* mRNA, thereby strengthening the immunosuppressive functions [55]. Interestingly, lactate inhibits oligomerization of MAVS, the adapter molecule for RLRs, and dampens RLR signalling [56]. The detailed mechanisms through which hypoxia affects IFN signalling in the iPSC macrophages will require further study. Our data suggest that hypoxia affects ISG expression by the disturbance of multiple pathways.

The increasing relevance to study arthropod-borne viruses due to the changing geographical location and transmission patterns associated with global warming, emphasises the importance to discover new therapeutic targets that may be broadly effective against diverse members of the *Flaviviridae* family [57]. In addition, virus infections and associated inflammation can affect local oxygen levels. Mitochondrial damage will increase reactive oxygen species, leading to a change in local metabolite availability and oxygen consumption [58–60]. Therapeutically targeting the pathways that dampen ISG expression could help treat a broad range of viral infections.

### Limitations of the study

The sample size of this study was limited by the production capacity of the embryoid body intermediates and lab closures during the SARS-CoV-2 pandemic. Both factors limited our ability to extend our findings by additional experiments such as genetic manipulation of the iPSC-derived macrophages or further measurements of ZIKV replication under hypoxic conditions.

## Supplementary Data

Supplementary material 1
