# Supplementary material for: Hypoxia dampens innate immune signalling at early time points and increases Zika virus RNA levels in iPSC-derived macrophages
Source: J Gen Virol. 2023 Aug 16;104(8):001885. doi: 10.1099/jgv.0.001885 (PMC10877081; doi:10.1099/jgv.0.001885)
Supplement: Supplementary material 1 [file jgv-104-1885-s001.pdf]

Supplementary Figures

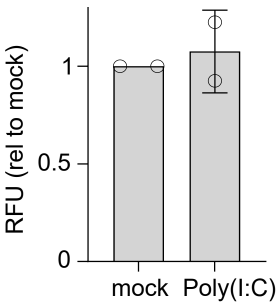

Suppl. Fig. 1: Poly(I:C) treatment does not affect cell viability.

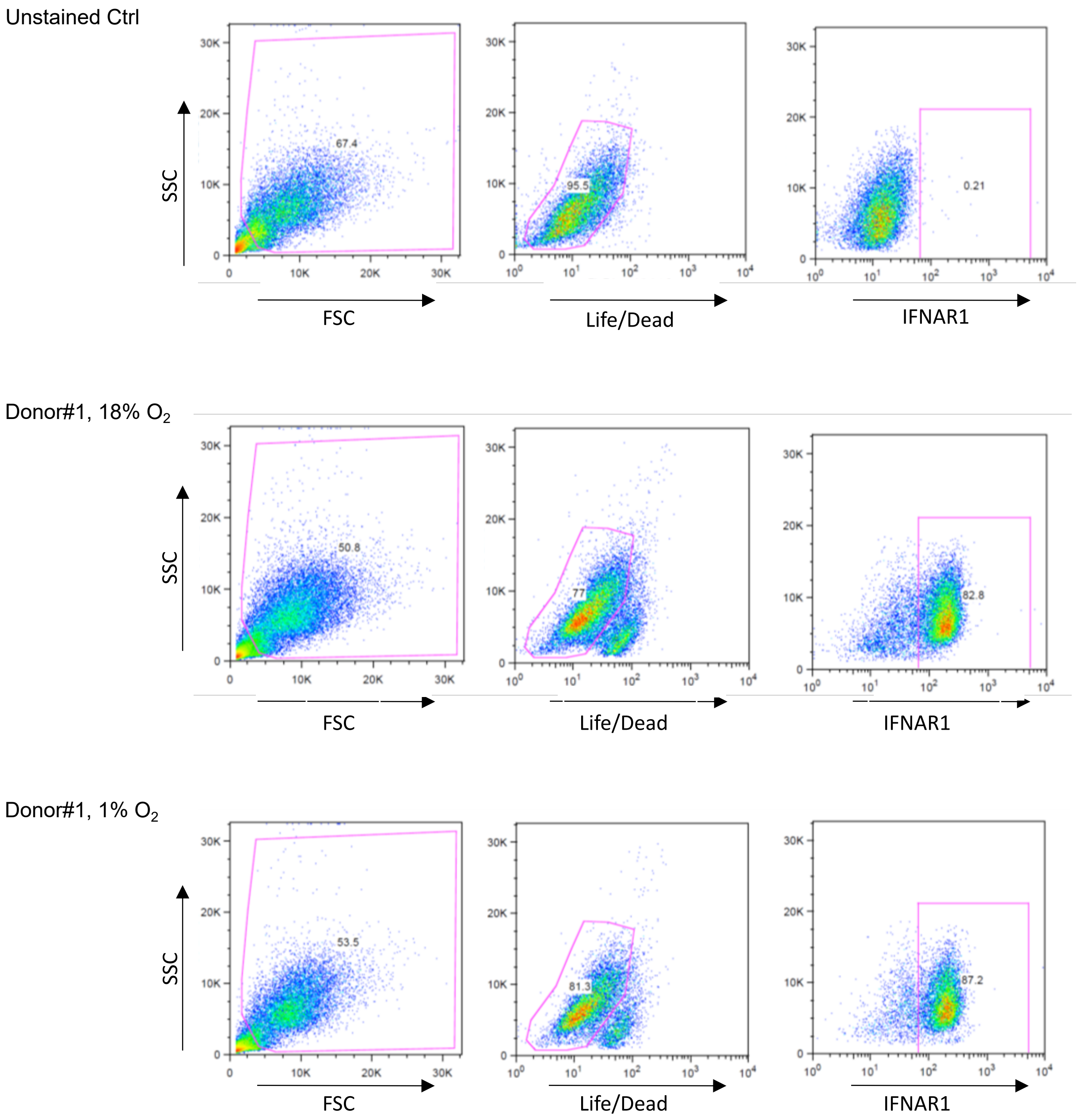

Suppl. Fig. 2: Gating strategy to stain for IFNAR1 surface expression on iPSC-derived macrophages.

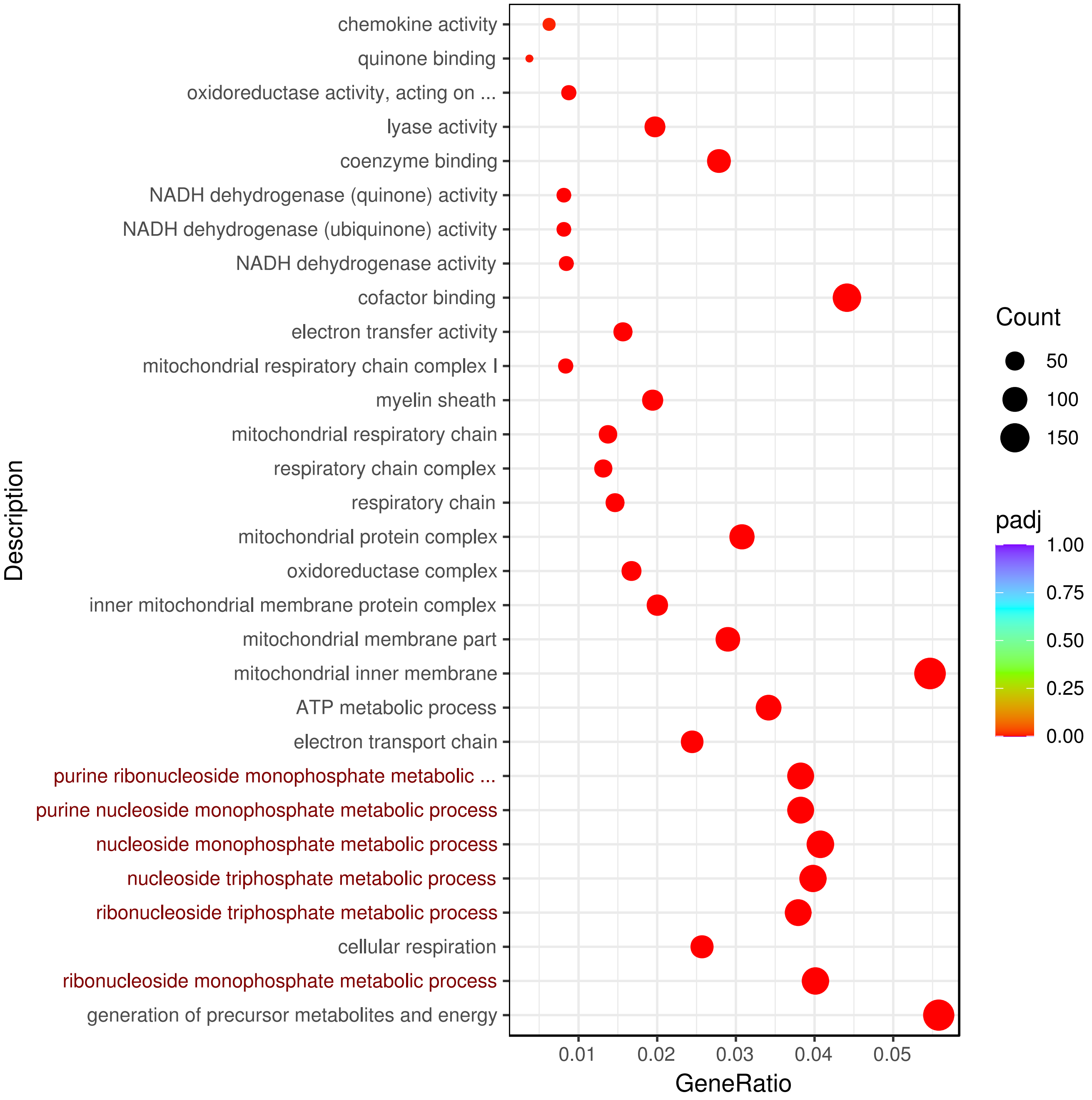

Suppl. Fig. 3: Hypoxia dysregulates nucleoside metabolism. .
